# Maturity-Onset Diabetes of the Young (MODY) Presenting With a Diabetic Foot Ulcer: A Case Report

**DOI:** 10.7759/cureus.95434

**Published:** 2025-10-26

**Authors:** Arfa Saleem, Falaknaz Saleem, Tooba Shaikh, Saroj Fatima, Halima Jahan Setu

**Affiliations:** 1 Internal Medicine, Baqai Institute of Diabetology & Endocrinology, Karachi, PAK; 2 Oncology, Jinnah Postgraduate Medical Centre, Karachi, PAK; 3 Acute and Internal Medicine, George Eliot Hospital NHS, Nuneaton, GBR; 4 Internal Medicine, George Eliot Hospital NHS, Nuneaton, GBR

**Keywords:** adolescent diabetes, diabetes treatment, difficult diagnosis, elevated hba1c, endocrinology and diabetes, maturity onset diabetes of the young, mody, type 1 and type 2 diabetes mellitus, type 1 diabetes mellitus (t1dm), type 2 diabetic mellitus (t2dm)

## Abstract

Maturity-onset diabetes of the young (MODY) is a rare form of diabetes that accounts for a minority of all reported cases of diabetes. Unlike the more common forms of diabetes, MODY typically presents in adolescents or young adults, usually before the age of 25. It is characterized by autosomal dominant inheritance and results from gene mutations involved in pancreatic beta-cell function. To date, 14 genetic subtypes have been identified, each with slightly different presentations and differing in their response to standardized treatment.

In this report, we highlight a case of a young female presenting with clinical features usually seen in chronic cases of type 2 diabetes mellitus, such as non-healing wounds on her foot, but who reported no other symptoms typically seen in diabetics. She was found to have raised fasting blood glucose levels on presentation as well as a strong family history of diabetes (early-onset diabetes in a first-degree relative). Upon testing, her glycated hemoglobin (HbA1c) level was also found to be 14.6% (the normal range for the HbA1c level is usually considered to be below 5.7%). The patient was later advised to undergo anti-GAD65 antibody testing, and a fasting C-peptide level was obtained to evaluate endogenous insulin production. Both tests returned within normal limits, suggesting preserved beta-cell function.

Given this history and atypical presentation, MODY was suspected. This case underscores the importance of considering MODY in young diabetic patients with significant family histories, as early genetic confirmation and personalized management can improve disease outcomes and lead to family screening.

## Introduction

A diabetic foot ulcer (DFU) is a break in the skin that extends into the dermis or deeper layers of tissue on a diabetic person's foot, commonly located on the bottom of the foot. It occurs due to a combination of neuropathy, disrupted blood supply, and often repetitive trauma in individuals with diabetes. Diabetes mellitus (DM) is an increasingly prevalent yet diverse chronic condition, encompassing a range of phenotypically distinct subtypes. Diabetes mellitus has two major subtypes: type 1 diabetes mellitus (T1DM) and type 2 diabetes mellitus (T2DM). T1DM typically leads to absolute insulin deficiency due to autoimmune processes and presentation in childhood or adolescence. T2DM, on the other hand, is characterized by insulin resistance with relative insulin deficiency, usually manifesting in adulthood, and is associated with obesity and metabolic syndrome. Among these are atypical forms of diabetes that lie within a poorly defined continuum between T1DM and T2DM. It is important to distinguish between the types of diabetes as the treatment plan differs for each type [1,2].

Maturity-onset diabetes of the young (MODY) is a rare form of diabetes. It is estimated that only 1-2% of all diabetics have this form. It is a distinct inherited disorder marked by the early development of diabetes (typically during adolescence or early adulthood), autosomal dominant transmission, absence of autoimmune destruction of pancreatic β-cells, and sustained C-peptide production. Due to the overlap of its clinical features with the more common forms of diabetes mellitus, it is often misdiagnosed as either type 1 or type 2 diabetes, which leads to an inappropriate treatment plan [2].

In this report, we discuss the presentation of MODY in a young female patient who was initially suspected to have type 2 diabetes due to her presentation with a diabetic foot ulcer.

## Case presentation

A 21-year-old female with no known comorbidities presented with non-healing wounds on her left upper foot for the past 2 weeks. Her history revealed corn formation near the fifth toe of the left foot, and near the big toe for the past two years, which developed spontaneously. Two weeks before the presentation, the patient tried a corn cap on her foot due to cosmetic reasons, which led to swelling, discoloration, and pus collection underneath the skin. The patient visited an outpatient clinic, where she was advised to use 2% fusidic acid cream as a topical antibiotic, which did not lead to any improvement. She then presented to the general medical ward.

Her personal history revealed that she was a student with no relevant occupational exposures and no history of trauma to the foot.

On clinical assessment, the patient was afebrile and normotensive with a normal heart rate; however, her fasting blood sugar was elevated to 237 mg/dL. Her body mass index was within the normal range. A HbA1c (glycated hemoglobin) test was arranged due to the clinical suspicion of diabetes.

**Figure 1:**
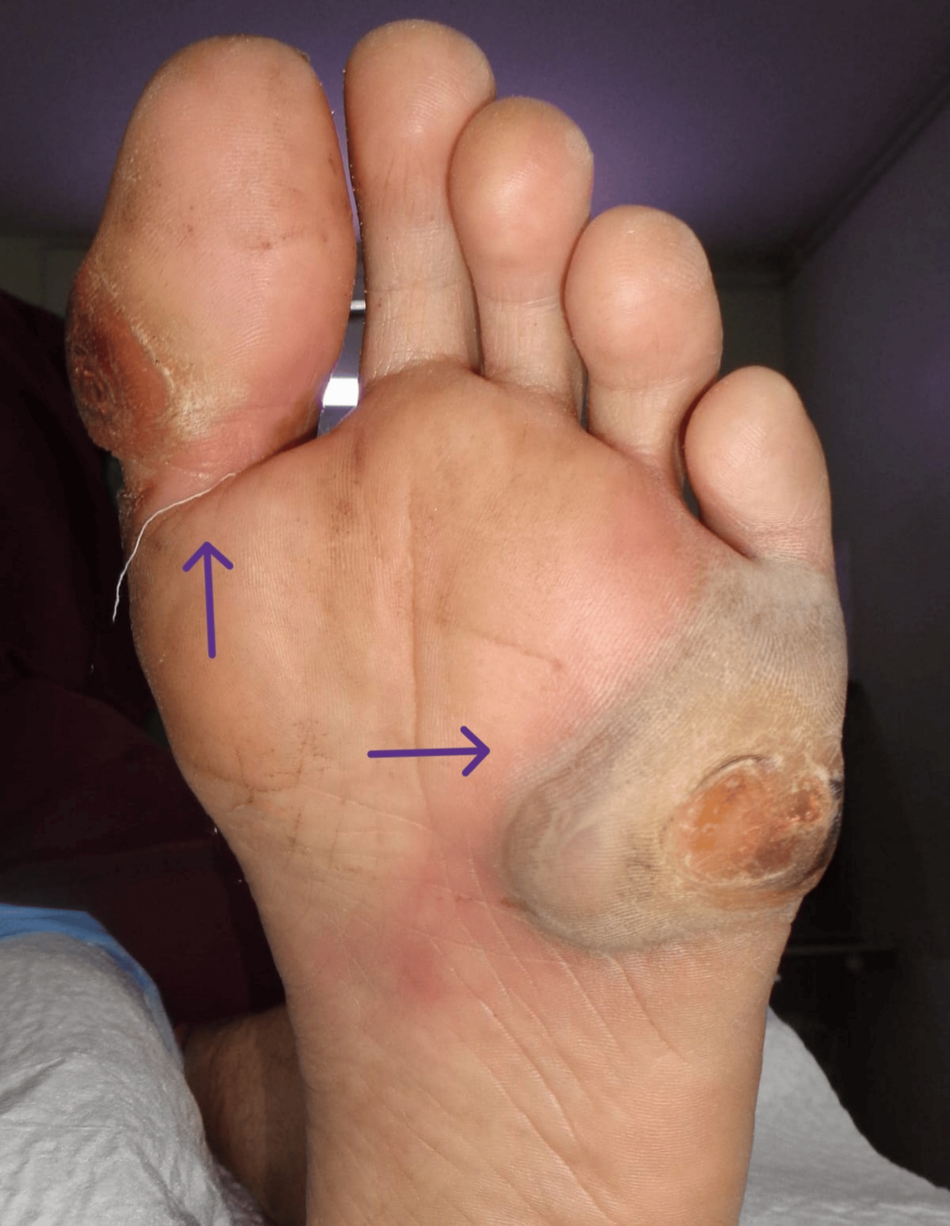
Left foot on presentation

Examination of the left foot revealed a 4x4 cm wound present on the plantar surface of the foot below the fifth toe, and a 2x2 cm wound near the big toe (Figure 1). The wounds were painful with no discharge.

On musculoskeletal examination, the patient had normal muscle bulk and tone in all extremities. Strength was 5/5 in all major muscle groups bilaterally. Monofilament testing did not show reduced sensation, and vibration sensation using a 128 Hz tuning fork was intact bilaterally. Peripheral vascular assessment revealed palpable dorsalis pedis and posterior tibial pulses bilaterally. Although the patient was a young adult with no vascular risk factors, an ankle-brachial index (ABI) was performed to rule out peripheral arterial disease as a contributing factor to ulcer formation. Assessment revealed an ABI of 1.0 on both sides, indicating no significant peripheral arterial disease. Examination of the other systems was unremarkable.

Her laboratory test results revealed no abnormalities except an elevated HbA1c level (Table 1).

**Table 1:** Laboratory investigations

Parameters	Normal range (units)	Patient results (on admission)
Hemoglobin	11.0-14.5 g/dL	12.4
Mean corpuscular volume	78.0-95.0 fL	85.2
White blood count	2.4-9.5 x 10^9^/L	7.2
Neutrophils	1.0-5.0 x 10^9^/L	3.0
Lymphocytes	1.2-4 x 10^9^/L	2.4
Eosinophils	0.15-0.5 x 10^9^/L	0.1
Monocytes	0.0-0.2 x 10^9^/L	0.1
Basophils	5 x 10^9^/L	0.0
Platelets	150-450 x 10^9^/L	196
Urea	20-40 mg/dL	29
Creatinine	0.8 to 1.2 mg/dl	0.5
Sodium	135-145 mmol/L	140
Potassium	3.5-5.1 mmol/L	4.2
Chloride	98-107 mmol/L	100
Bicarbonate	22-29 mmol/L	26
HbA1c (glycated hemoglobin A1c)	< 5.7%	14.6%

Her HbA1c level was found to be 14.6%. A normal HbA1c percentage is considered to be below 5.7% (the reference interval for HbA1c in a nondiabetic adult or child is 4.0-5.7%).

On a detailed systemic review, the patient did not report increased thirst, increased urination, burning micturition, or blurred vision. There was no recent weight loss. A fundoscopy revealed no abnormalities.

A family history revealed a pattern of early-onset diabetes. The patient’s mother was diagnosed as a diabetic at 25 years of age and was started on subcutaneous insulin, and her maternal uncle was diagnosed as a diabetic at the age of 35 years. Her late maternal grandparents were also diagnosed as diabetics; however, the exact age of presentation was unknown, and specific details regarding the diabetes type and treatment were not available.

The patient was initiated on a subcutaneous insulin regimen during her hospital stay, consisting of Humulin R (regular insulin) 6 units and Humulin N (NPH insulin) 14 units in the morning, and Humulin R 4 units with Humulin N 10 units in the evening. Blood glucose levels were monitored regularly during admission, and an improvement was noted, with values maintained around 150-160 mg/dL on this regimen. At discharge, she was advised to continue the same subcutaneous insulin regimen until her next outpatient follow-up visit.

She was also advised to undergo incision and drainage of her left foot wounds by the surgical team, and the procedure was carried out on day 2 of her admission. Intravenous antibiotics were commenced according to hospital protocol, including sulbactam/cefoperazone 2 g twice daily and teicoplanin 200 mg once daily, which were continued for five days. No X-rays or advanced imaging were performed to evaluate for deeper infection during this admission.

Upon discharge, further investigations were advised to determine the type of diabetes mellitus the patient was suffering from. The investigations were planned after discharge due to the patient's financial limitations, as they preferred to have the testing done at another on-site laboratory.

The patient was advised anti-GAD65 antibodies to assess for autoimmune diabetes (T1DM). Additionally, a fasting C-peptide level was obtained to evaluate endogenous insulin production. Both tests returned within normal limits, suggesting preserved beta-cell function and reducing the likelihood of autoimmune-mediated diabetes (T1DM) (Table 2) [3-9].

**Table 2:** Glutamic acid decarboxylase 65 antibody assay and serum C-peptide levels

Parameters	Normal range	Patient values
Parameters	Normal range (units)	Patient results
Serum glutamic acid decarboxylase antibody (GAD 65) assay	<10 IU/ml	<5.0
Serum C-peptide	1.1-4.4	1.61

The above findings were suggestive of this being a presentation of MODY. While MODY usually presents with only a mild fasting hyperglycemia, and foot ulcers are a very rare complication, in this case, it was suspected due to the lack of systemic symptoms usually seen in T2DM, and the earlier age of presentation, normal BMI, and negative autoimmune markers. The maternal family history of early-onset diabetes also supported this suspicion. Although genetic testing is the gold standard for confirming MODY, it was not performed due to financial constraints, which remain a common limitation in resource-limited settings.

On follow-up in the outpatient clinic, 15 days after incision and drainage, the patient’s insulin regimen was discontinued, as her blood glucose levels had stabilized. After specialist consultation, she was initiated on a trial of oral sulfonylureas (gliclazide 5 mg once daily), and regular weekly follow-up assessments were advised to monitor glycemic response. The foot wound was improving at that time, although not yet fully resolved. The sulfonylurea trial was empiric, based on the clinical presentation, history, and laboratory results, as genetic subtype confirmation of MODY (e.g., HNF1A or HNF4A mutations) was not available. Additionally, she was advised to maintain a home blood sugar chart to track daily glucose levels and was provided with dietary and lifestyle modification advice.

At the subsequent follow-up visit, her blood glucose levels were maintained below the threshold range on sulfonylureas, and the wound continued to improve. Repeat HbA1c testing had not yet been performed at the last follow-up due to financial limitations. Lifestyle advice was reinforced.

## Discussion

MODY is a monogenic form of diabetes that runs in families. It is rare, accounting for just 1%-2% of all diagnosed cases of diabetes, and it is frequently misclassified as either type 1 or type 2 diabetes [1]. MODY presents a significant diagnostic challenge due to a variety of clinical presentations associated with MODY, including asymptomatic patients with only elevated blood glucose levels revealed on testing, to patients presenting with symptoms classically seen in more common forms of diabetes, such as frequent urination, increased thirst, dehydration, and recurrent skin infections. As a result, MODY is often misdiagnosed as either T1DM or T2DM. Current standards recommend considering monogenic diabetes when features such as young onset, non-obesity, negative autoantibodies, and preserved C-peptide are present [1-4]. In our patient, her young age, normal BMI, negative autoimmune markers, and preserved C-peptide, together with a multigenerational history, raised our suspicion for MODY over T1DM/T2DM.

However, it is crucial to distinguish MODY from type 1 and type 2 diabetes because the standardized treatments are different [3-14]. Furthermore, MODY results from single-gene mutations, with first-degree relatives carrying a 50% likelihood of inheriting the same pathogenic variant. Consequently, an early diagnosis allows for better management, improved prognosis, and the opportunity to provide appropriate counseling and screening for relatives.

To date, there are mutations in at least 14 different genes that result in the MODY phenotype. Depending on the genetic etiology, the various genetic subtypes differ in terms of age of onset, pattern of hyperglycemia, response to treatment, and extra-pancreatic manifestations. Most cases of MODY are due to mutations in three genes, hepatic nuclear factor 1 alpha (HNF1A), hepatic nuclear factor 4 alpha (HNF4A), and glucokinase (GCK), with a detection rate that varies among different study populations.

Treatment varies between genetic subtypes and differs markedly from the management of type 1 and type 2 diabetes. Patients with HNF1A- and HNF4A-MODY often respond well to sulfonylureas and can discontinue insulin, whereas GCK-MODY generally requires no pharmacologic therapy (Table 3). For this reason, clinical suspicion of MODY should remain in the physician’s mind when seeing patients presenting with hyperglycemia, especially in those under 30 years of age, both in primary and secondary care [4-13].

**Table 3:** Genetic mutations associated with maturity-onset diabetes of the young (MODY)

Gene	Prevalence amongst those with MODY	Other clinical features
HNF1A (MODY3)	30%–60%^*^	This shows high penetrance. Hyperglycemia is progressive along with progressive β-cell failure. Responsive to sulphonylureas.
GCK (MODY 2)	30%–50%^*^	Raised fasting glucose levels, approximately in the range of 5.5–8 mmol/L. Generally does not require treatment.
HNF4A (MODY 1)	5%-10%	Presents in a similar manner to HNF1A mutations. Can present during the neonatal period as transient or persistent neonatal hypoglycaemia. Gradual progressive β-cell failure leading to reemergence of diabetes in adolescence. Responsive to sulphonylureas.

In our case, hepatic nuclear factor 1 alpha (HNF1A) MODY was clinically suspected, and hence the patient was initiated on a trial of sulphonylureas, with discontinuation of the insulin regimen.

A correct diagnosis is linked to important treatment benefits, as it drives the choice of the best treatment, with most patients responding well to oral hypoglycemics, depending upon the gene mutations [3,13]. Correct diagnosis would also improve quality of life by reducing both treatment and financial burden and avoiding unnecessary insulin injections. The right treatment plan may help in preventing future complications, such as the diabetic foot ulcer we observed in our patient, as well as the more feared complications of diabetes like retinopathy, chronic kidney disease, and ischemic heart disease.

In countries like Pakistan, access to genetic testing is often limited, and the pattern of MODY gene variants may differ from that seen in European populations. This highlights the importance of relying on clinical features to identify possible MODY cases when genetic tests are not easily available [15].

An early diagnosis will also lead to genetic counseling for the relatives and offspring; where financial constraints are a factor, cost-effective screening methods for family members may be advised, such as fasting blood glucose levels monitored at home and shown to healthcare providers, and arranging HbA1c testing for all first-degree relatives under 35 years of age, followed by genetic counseling if indicated. Proper education for the patient and their family members remains essential to ensure understanding of the condition and adherence to follow-up plans [12,14].

## Conclusions

Maturity-onset diabetes of the young (MODY) is frequently misdiagnosed as type 1 or type 2 diabetes. This leads to inappropriate treatment plans, unnecessary insulin use, and avoidable complications due to delayed initiation of effective oral agents. In this case, the patient presented with a diabetic foot ulcer, which is a common complication of long-standing type 2 diabetes but represents an atypical manifestation of MODY, likely reflecting prolonged undiagnosed hyperglycemia.

Clinicians should maintain a high index of suspicion for MODY in young, non-obese patients with preserved C-peptide, negative autoimmune markers, and a strong family history of early-onset diabetes. Genetic testing should be offered when feasible; however, in resource-limited settings, clinical judgment remains crucial for reducing complications and improving long-term outcomes. By presenting this case, we aim to emphasize the importance of early recognition of MODY to support the timely initiation of personalized treatment and to prevent future complications.
